# Prevalence of Mupirocin and Methicillin-Resistant Staphylococcus aureus in Nasal Carriage Among Healthcare Workers in an Intensive Care Unit and Post-decolonization Screening Outcomes at a Tertiary Care Hospital: A Prospective Study

**DOI:** 10.7759/cureus.46435

**Published:** 2023-10-03

**Authors:** Bavani Premanand, Sabarinathan Thiyagarajan, Swarnalingam Thangavelu, Saleem Mohammed Ali, Fatima Shirly Anitha George

**Affiliations:** 1 Microbiology, Melmaruvathur Adhiparasakthi Institute of Medical Sciences and Research, Chengalpattu, IND; 2 Anesthesiology and Critical Care, Melmaruvathur Adhiparasakthi Institute of Medical Sciences and Research, Chengalpattu, IND; 3 Developmental Pediatrics, NHS Foundation Trust Hospital, Surrey, GBR

**Keywords:** methicillin-resistant staphylococcus aureus, methicillin-resistant coagulase-negative staphylococcus, mupirocin-resistant staphylococcus aureus, mupirocin-resistant coagulase-negative staphylococcus, post-decolonization, low level mupirocin resistance, high level mupirocin resistance, nasal carrier, intensive care unit, healthcare workers

## Abstract

Introduction: Nasal carriage of *Staphylococcus* species plays an important role in the epidemiology and pathogenesis of both community and healthcare-associated infections. Coinciding the emergence of methicillin-resistant *Staphylococcus aureus* (MRSA) is a challenge for clinicians to prevent their spread. Mupirocin is a topical antimicrobial agent approved for eradicating nasal carriage of staphylococcal species in adult patients and healthcare workers (HCWs). The increasing prevalence of mupirocin resistance among *Staphylococcus aureus* and coagulase-negative staphylococci species could be an important threat to the future use of mupirocin against MRSA.

Objective: The aim of this study is to determine the prevalence of MRSA from nasal swabs of HCWs in intensive care units and its level of resistance pattern of mupirocin in all isolates of *Staphylococcus* species by disk diffusion and epsilometer test (E-test) and to determine post decolonization screening.

Materials and methods: A total of 67 HCWs (doctors, nursing staff, technicians, and housekeeping staff) in the medical and surgical intensive care units were included in the study. Nasal swabs were collected from the subjects and cultured onto nutrient and blood agar, which were then incubated at 37ºC for 18 to 24 hours. *Staphylococcus aureus* and coagulase-negative*Staphylococcus* species (CoNS) were identified by standard biochemical techniques. Methicillin resistance was detected by the disk diffusion method using a 30 µg cefoxitin disk as per the Clinical and Laboratory Standards Institute (CLSI) guidelines, and mupirocin resistance was detected using a 5 µg mupirocin disk. The resistance strains were further subjected to E-strip testing to determine the level of mupirocin resistance.

Results: A total of 72 isolates were grown from the 67 subjects used in this study. Nine strains (12.5%) grew *S. aureus,* and 52 strains (72.2%) grew CoNS. Methicillin resistance was seen in five isolates (6.9%) of *S. aureus* and 45 isolates (62.5%) of CoNS. Mupirocin resistance was seen in 11 isolates of methicillin-resistant coagulase-negative *Staphylococcus* species (MRCoNS), where three isolates (4.1%) showed low-level mupirocin resistance MuL and eight isolates (11.11%) showed high-level mupirocin resistance MuH. None of the isolates of MRSA, methicillin-sensitive *Staphylococcus aureus* (MSSA), and methicillin-sensitive coagulase-negative *Staphylococcus*species (MSCoNS) were resistant to mupirocin. Seven out of nine HCWs (77.8%) showed clearance of the organism after decolonization therapy.

Conclusion: The prevalence of emerging resistance to mupirocin in MRSA and MRCoNS is of great concern, especially in the nasal carrier state of HCWs. Hence, methicillin and mupirocin resistance in *S. aureus* and CoNS must be detected in HCWs as a routine protocol, and decolonization measures should be undertaken to prevent healthcare-associated infections.

## Introduction

*Staphylococcaceae* are a ubiquitous family of gram-positive cocci that are natural habitants of skin, mucosa, and anterior nares. They are grouped into coagulase-negative *Staphylococcus* (CoNS) and coagulase-positive *Staphylococcus*, depending on their ability to produce the coagulase enzyme. Despite being significant commensal colonizers of both animals and people, staphylococcal species have been linked to a number of illnesses, including abscesses, septicemia, meningitis, pneumonia, and toxicosis [1]. Staphylococci are a major cause of infections in hospital and community settings. Therefore, the emergence of methicillin-resistant staphylococci, including methicillin-resistant *Staphylococcus aureus* (MRSA) and methicillin-resistant coagulase-negative *Staphylococcus* (MRCoNS), poses a significant public health risk worldwide.

The main sources of MRSA infections are blood, pus, ear discharge, and nasal and throat swabs. MRSA and MRCoNS are recognized for their ability to exhibit resistance to all antibiotics belonging to the beta-lactam group, which leads to increasing treatment failure in clinical practice and thereby to high fatality rates [2]. Nasal carriage of* Staphylococcus aureus* plays a key role in the epidemiology and pathogenesis of infection and is a major risk factor for both community-acquired and healthcare-associated infections [3]. MRSA spreads readily in hospitals from carriers or infected persons. Colonized healthcare workers (HCWs) are generally asymptomatic, though they are a potential reservoir and disseminator of MRSA in hospitals [4].

CoNS has a major role in causing device-associated infections and infections among immunocompromised patients. Multidrug-resistant phenotypes are more common among CoNS compared to *S. aureus*. Methicillin resistance in CoNS may not only lead to treatment failure but also spread this resistance to *S. aureus,* which may pose a challenge to clinicians [5]. When MRSA was identified in 1960, it was shown to harbor a causal mecA gene encoding a modified penicillin-binding protein (PBP) designated PBP2a, that has low affinity for beta-lactam antibiotics and confers methicillin resistance to staphylococci [6].

Infection prevention strategies such as nasal decolonization are employed to minimize the occurrence of staphylococcal infection and reduce the risk of transmission within healthcare settings [7]. The Infectious Diseases Society of America (IDSA) practice guidelines for skin and soft tissue infections and the CDC guidelines recommend intranasal mupirocin as a decolonization regimen for MRSA carriers [8].

Mupirocin (pseudomonic acid A), derived from* Pseudomonas fluorescens*, has been in clinical use since 1985. It binds to a bacterial isoleucyl transfer RNA (tRNA) synthetase enzyme, which is encoded by the ileS gene and inhibits protein synthesis. Due to the prolonged and widespread use of this medicine, mupirocin-resistant organisms have emerged [9].

Mupirocin-resistant strains are grouped into two distinct categories: strains with low-level resistance (MuL) showing minimum inhibitory concentration (MIC) of 8-256 µg/ml and strains with high-level resistance (MuH) having MIC ≥ 512 µg/ml. Susceptible strains are defined as having MIC ≤ 4 µg/ml. Both low-level MuL and high-level MuH resistance of mupirocin have been reported due to its increased use. Low-level resistance is due to the mutational change in the chromosomally coded ileS-2 mupA gene [10]. High-level resistance is the acquisition of plasmid containing the mupA gene encoding an additional isoleucyl tRNA synthetase enzyme. It is attributed to another gene mupB. High-level mupirocin resistance in *S. aureus* is a serious clinical problem linked to decolonization failure, particularly in MRSA carriers [11]. It has been suggested that mupirocin-resistant CoNS might be an important source of mupA determinant in MRSA. The increasing prevalence of transferable mupirocin resistance among CoNS species could be an important threat to the future use of mupirocin against MRSA [12].

It is important to identify MRSA carriers among HCWs, especially those who work in intensive care units, as these individuals could potentially infect their patients leading to healthcare-associated infections and thereby causing an extended hospital stay. MRSA screening is done to prevent its spread to HCWs. The data in the literature are limited with regard to the sensitivity of the drug mupirocin and the level of resistance in MRSA and MRCoNS carriers. Hence, this study was undertaken to analyze the extent of mupirocin resistance among HCWs with MRSA and MRCoNS carriers in the resource-limited setting of the present study.

Objectives

To determine the prevalence of MRSA from the nasal swabs of HCWs in intensive care units and the prevalence of mupirocin resistance in all isolates of MRSA, methicillin-sensitive *Staphylococcus aureus* (MSSA), and CoNS by disk diffusion and epsilometer test method. The level of resistance pattern of mupirocin in *S. aureus* and CoNS isolates was also analyzed. Post-decolonization screening for clearance among mupirocin-resistant MRSA and MRCoNS carriers was also determined.

## Materials and methods

This prospective cross-sectional study was conducted in the intensive care unit of a tertiary medical college hospital from May 2022 to July 2022.

Inclusion and exclusion criteria

All HCWs (doctors, nursing staff, housekeeping staff, and technicians) of medical and surgical intensive care units were included in the study. HCWs with upper respiratory tract infections were excluded from the study.

The age, sex, designation, duration of working in the critical care unit, and other relevant information were obtained in the data collection proforma. Informed consent from participants was obtained. The Institutional Ethics Committee (Human Studies), Melmaruvathur Adhiparasakthi Institute of Medical Sciences and Research issued approval (MAPIMS/IEC/52/2022) in May 2022.

Sample collection

Nasal swabs from all HCWs were collected from both nostrils by approximately inserting the sterile cotton swab 2 cm into the anterior nares pre-wetted with sterile saline and rotating in the anterior nasal mucosa for three seconds, and using the same swab, this was repeated on another nostril. Swabs were then transported to the microbiology laboratory in the transport tube streaked on blood agar and nutrient agar and incubated at 37ºC for 18 to 24 hours. Identification of *S. aureus* and CoNS was done by standard biochemical techniques. Identification of methicillin resistance was done using cefoxitin disk (CX) 30 µg by the disk diffusion method as per the Clinical and Laboratory Standards Institute guidelines.

Preliminary screening of mupirocin resistance of all isolates was done using a 5 µg mupirocin disk using the disk diffusion method. Mueller Hinton agar plates were lawn cultured with test strains and a 5 µg mupirocin disk was placed. A zone size of ≥ 14 mm was considered as mupirocin sensitive and a zone size of ≤ 12 mm was considered as mupirocin-resistant strain. The minimum inhibitory concentration (MIC) for mupirocin resistance was determined by the E-test method with an antibiotic concentration gradient range of 0.064 µg/ml to 1024 µg/ml. The test was performed and interpreted according to the manufacturer’s recommendations. MIC falling between 8 and 256 µg/ml was taken as low-level mupirocin resistance MuL and strains with MIC ≥ 512 µg/ml were considered as high-level mupirocin resistance MuH. Strains with MIC ≤ 4 µg/ml were considered mupirocin-susceptible strains.

Decolonization method

As per the CDC guidelines [8], the decolonization protocol consisted of intranasal application of 2% mupirocin ointment twice daily and chlorhexidine bath daily for one week for all mupirocin-sensitive MRSA, MRCoNS, and low-level mupirocin-resistant MRSA and MRCoNS carriers. Intranasal application of neomycin ointment twice daily for one week was advised for high-level mupirocin-resistant MRSA and MRCoNS carriers [12]. All colonized HCWs were given counseling and instructions to follow the decolonization regimen.

Re-swabbing and screening were done for mupirocin-resistant MRSA and MRCoNS carriers after 12 weeks of post-decolonization therapy [13].

Statistical analysis

In our study, we recorded the data in Microsoft Excel 2019 (Microsoft Corporation, Redmond, WA) and SPSS software version 20.0 (IBM Corp., Armonk, NY) was used to analyze the data. Frequencies and percentages of the organisms isolated were tabulated. The association between the professional category and different organisms isolated was calculated using the chi-square test. A p-value of <0.05 was considered as significant.

## Results

**Figure 1:**
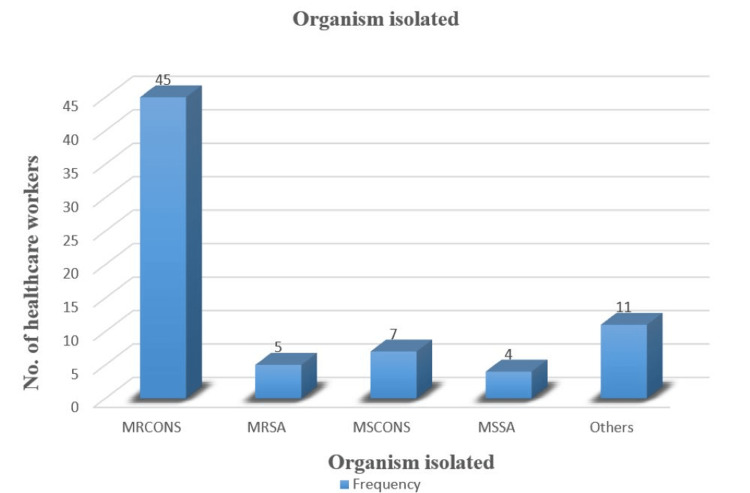
Prevalence of different Staphylococcus species among 67 healthcare workers MRCoNS: methicillin-resistant coagulase-negative *Staphylococcus*; MRSA: methicillin-resistant *Staphylococcus aureus*; MSCoNS: methicillin-sensitive coagulase-negative *Staphylococcus*; MSSA: methicillin-sensitive *Staphylococcus aureus*.

In our study, among the 67 HCWs working in intensive care units, consecutive non-repetitive organisms isolated were 72. Out of which, *S. aureus* was isolated in nine (12.5%), and CoNS were isolated in 52 (72.2%) HCWs. MSSA was colonized in four (5.6%) and methicillin-sensitive CoNS (MSCoNS) were colonized in seven (9.7%) HCWs. Methicillin resistance was seen in five isolates (6.9%) of *S. aureus* and 45 (62.5%) isolates of CoNS. Other organisms isolated from the nasal carriage of HCWs were 11 (15.3%) (Table 1 and Figure 1).

**Table 1:** Culture results of nasal swabs of 67 healthcare workers MRCoNS: methicillin-resistant coagulase-negative *Staphylococcus*; MRSA: methicillin-resistant *Staphylococcus aureus*; MSCoNS: methicillin-sensitive coagulase-negative *Staphylococcus*; MSSA: methicillin-sensitive *Staphylococcus aureus.*

Organism grown	Frequency	Percentage
MRCoNS	45	62.5
MRSA	5	6.9
MSCoNS	7	9.7
MSSA	4	5.6
Others	11	15.3
Total	72	100.00

With respect to the professional category, the highest prevalence of nasal colonization of *S. aureus* and CoNS was observed among nursing staff (73.33%), followed by technicians (15.55%), while doctors had the lower prevalence of nasal colonization (11.11%) among the HCWs. The nasal colonization of MRCoNS among the nursing staff and technicians and also colonization of MRSA among doctors, technicians, and housekeeping staff were statistically significant (p < 0.05) (Table 2).

**Table 2:** Nasal carriage of Staphylococcus species among different categories of healthcare workers MRCoNS: methicillin-resistant coagulase-negative *Staphylococcus*; MRSA: methicillin-resistant *Staphylococcus aureus*; MSCoNS: methicillin-sensitive coagulase-negative *Staphylococcus*; MSSA: methicillin-sensitive *Staphylococcus aureus*.

Category wise	Frequency	Percentage	P-value
Doctors: MRCoNS	5	71.4	0.104
MSCoNS	2	28.6	0.001
MRSA	0	0.0	0.001
MSSA	0	0.0	0.001
Others	0	0.0	0.001
Nursing staff: MRCoNS	33	66.0	0.001
MSCoNS	4	8.0	0.229
MRSA	3	6.0	0.301
MSSA	4	8.0	0.229
Others	6	12.0	0.134
Housekeeping: MRCoNS	0	0.0	0.315
MSCoNS	0	0.0	0.001
MRSA	0	0.0	0.001
MSSA	0	0.0	0.001
Others	2	100.00	0.001
Technicians: MRCoNS	7	53.8	0.005
MSCoNS	1	7.7	0.001
MRSA	2	15.4	0.001
MSSA	0	0.0	0.001
Others	3	23.1	0.001

**Figure 2:**
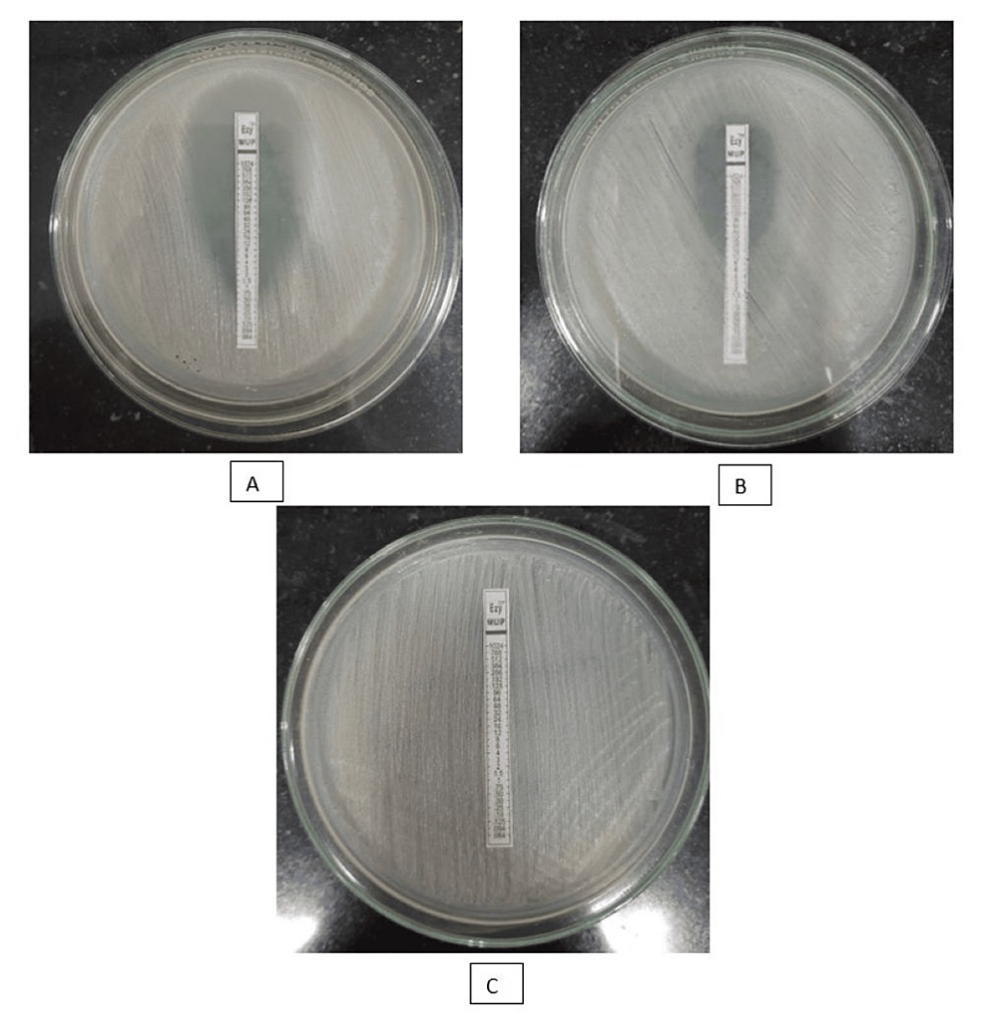
Mueller Hinton agar showing the level of mupirocin resistance of methicillin-resistant coagulase-negative Staphylococcus using mupirocin E-strip A: mupirocin susceptible; B: low-level mupirocin resistance MuL; C: high-level mupirocin resistance MuH.

In our study, out of 72 organisms isolated among 67 HCWs, which included the isolates of MRSA, MSSA, and MSCoNS, 100% were sensitive to mupirocin. But three isolates (4.1%) of MRCoNS showed low-level mupirocin resistance and eight isolates (11.11%) showed high-level resistance to mupirocin drug (Table 3 and Figure 2).

**Table 3:** Mupirocin resistance in Staphylococcus species MuL: low-level mupirocin resistant; MuH: high-level mupirocin resistant; MRSA: methicillin-resistant *Staphylococcus aureus*; MRCoNS: methicillin-sensitive coagulase-negative *Staphylococcus.*

Mupirocin resistance					
Category	MRSA - 5		MRCoNS - 45		Total N = 72, (N/%)
	MuL (N/%)	MuH (N/%)	MuL (N/%)	MuH (N/%)	
Doctors	0	0	1 (1.3%)	1 (1.3%)	2 (2.6%)
Nursing staff	0	0	2 (2.8%)	4 (5.5%)	6 (8.3%)
Technicians	0	0	0	3 (4.2%)	3 (4.2%)
Housekeeping	0	0	0	0	0
Total	0	0	3 (4.1%)	8 (11.11%)	11 (15.28%)

In our study, after 12 weeks of decolonization, re-swabbing from anterior nares and screening for clearance were done for nine HCWs who were found to have high-level and low-level mupirocin resistance in MRCoNS carriers. Two out of 11 HCWs who were found to be mupirocin-resistant MRCoNS carriers had left the work. Among the nine HCWs of mupirocin resistance MRCoNS carriers after decolonization treatment, seven HCWs (77.8%) showed clearance of the organism, and two HCWs (22.2%) showed colonization with MRCoNS.

## Discussion

HCWs play an important role in the epidemiology and pathogenesis of healthcare-associated infection by harboring the resistant strains of bacteria that spread in the hospitals. Hence, detection of MRSA and MRCoNS colonization among HCWs in hospitals is important, especially for those working in intensive care units who act as potential sources of infection to their immunocompromised patients.

In our study, the prevalence of *S. aureus* in the nasal carriage of HCWs was 12.5% and that of CoNS was 72.2%, which is almost similar to a study conducted in 2013 that found 17.5% of *S. aureus* and 73% of CoNS colonizing the nasal carriage of HCWs [14]. In contrast to this, another study found 63% of *S. aureus* and 22% of CoNS isolated among HCWs [15]. Agarwal et al. observed 32.8% of HCWs colonized with CoNS in their study [5].

The findings of MRSA and MRCoNS among HCWs were 6.9% and 62.5% in our study, which was marginally comparable with other studies like Kaur et al., who observed 14.28% of MRSA and 24.29% of MRCoNS colonized among HCWs, and Oommen et al., who observed 28.7% of MRSA and 23.3% of MRCoNS colonized among HCWs [12,16]. In contrast, another study conducted in 2009 found only one HCW (1.8%) was colonized with MRSA [17]. Also, Agarwal et al. observed only 7.6% of HCWs were colonized with MRCoNS [5]. In our investigation, HCWs in the intensive care unit had considerably higher MRCoNS colonization rates than MRSA colonization rates.

With respect to the professional category, in our study, the prevalence of colonization of *Staphylococcus* species was higher among nursing staff (73.33%), followed by technicians (15.55%). Doctors presented with a lower prevalence of nasal colonization of *Staphylococcus* species (11.11%). This is consistent with other studies in which a higher prevalence of colonization was found among nursing staff (47.37% and 55%), followed by doctors (21.05% and 20%), respectively [14,16]. In contrast to this, Rongpharpi et al. showed a higher prevalence of colonization among doctors (25.7%), followed by nursing staff (22.86%) [18].

In our study, none of the doctors were MRSA carriers, which is on par with a study conducted in 2021 [15]. However, doctors were colonized with MRCoNS in our study (71.4%). At the same time, the prevalence of MRCoNS was higher among nursing staff (66%) and technicians (53.8%) than MRSA colonization, and it was also statistically significant (p < 0.005). This is in contrast to the study carried out in 2016 where no doctors, nursing staff, and technicians were colonized with MRCoNS [5]. Higher nasal carrier rates of MRSA and MRCoNS among nursing staff and technicians may be attributed to their spending long hours in the hospital, direct patient contact, and also associated with their personal hygiene.

Mupirocin is the mainstay in the decolonization regimen to prevent healthcare-associated staphylococcal infections and also various skin and soft tissue infections. However, prolonged indiscriminate use of mupirocin can lead to the development of mupirocin resistance. In our study, among the MRCoNS colonization in HCWs, three isolates (4.1%) of MRCoNS showed low-level mupirocin resistance and eight isolates of MRCoNS (11.11%) showed high-level resistance to mupirocin. Interestingly, all isolates of MRSA, MSSA, and MSCoNS among HCWs were 100% susceptible to mupirocin in our study. Similarly, Kaur et al. also showed five isolates of MRCoNS were high-level mupirocin-resistant, and among two isolates of MRSA, one was low-level and the other was high-level resistant to mupirocin, and all isolates of MSSA and MSCoNS were 100% susceptible to it in their study [16]. Agarwal et al. also showed three isolates of MRCoNS were resistant to mupirocin [5].

Mupirocin demonstrates higher efficacy with a significant duration of nasal clearance of MRSA in carriers. Mupirocin resistance, especially high-level mupirocin resistance, provides very less treatment options. The presence of higher rates of mupirocin resistance among CoNS is also a concern since the gene encoding for mupirocin resistance mupA gene can be transferred from CoNS species to MRSA, which is an important threat to mupirocin usage against MRSA in the future [12].

High-level mupirocin resistance has been associated with failure to clear the organism in carriers on mupirocin therapy, whereas low-level mupirocin-resistant carriers can be controlled with mupirocin therapy with an ointment that contains mupirocin concentration of 2000 µg/ml, which is much higher than the low-level mupirocin-resistant MIC [19]. Detection of MRSA and MRCoNS in an HCW can be treated with seven-day chlorhexidine-based baths and topical 2% mupirocin ointment along with reallocation or absence from duty until two negative culture reports are documented. Fusidic acid and neomycin cream should be considered if colonization persists after two courses of mupirocin or if the nasal swab confirms high-level mupirocin resistance [12,15].

Education

In our study, all HCWs were educated and counseled about MRSA risk factors, route of transmission, outcomes associated with infection, and effective preventive measures, including behavioral changes such as proper hand hygiene, environmental cleaning, and disinfection.

In the present study, decolonization was performed for HCWs who were colonized with MRSA and MRCoNS. Intranasal 2% mupirocin twice daily along with chlorhexidine bath daily for one week was advised for mupirocin-sensitive MRSA, MRCoNS, and low-level mupirocin resistance MRCoNS carriers. Intranasal neomycin ointment twice daily for one week was recommended for high-level mupirocin resistance MRCoNS carriers. In our study, we also performed post-decolonization screening to determine the clearance of MRSA and MRCoNS in nasal carriers of HCWs, to know the outcome of decolonization.

We found that after decolonization treatment in our study, seven out of nine HCWs (77.8%) who were mupirocin-resistant MRCoNS carriers were shown clearance of organism, and two out of nine HCWs (22.8%) were colonized with MRCoNS. These two HCWs were advised for another course of decolonization treatment.

Limitation

The main limitation of our study would be the small sample size.

## Conclusions

Overall, in the present study, the nasal colonization of MRCoNS among HCWs was significantly higher than MRSA. Mupirocin resistance was seen higher among MRCoNS isolates. Both low and high-level mupirocin resistance was seen. It is advisable for the infection control committee to routinely perform nasal decolonization among HCWs to prevent the spread of infections from colonized HCWs to the immunocompromised patients and co-workers and to find mupirocin resistance among the carrier strains so that successful decolonization will be achieved with proper dosage of mupirocin and alternate decolonization methods may be used if needed. Compliance with the decolonization protocol was also associated with rapid clearance of MRSA colonization.

## References

[1] Dilnessa T, Bitew A (2016). Prevalence and antimicrobial susceptibility pattern of methicillin resistant Staphylococcus aureus isolated from clinical samples at Yekatit 12 Hospital Medical College, Addis Ababa, Ethiopia. BMC Infect Dis.

[2] Vandenesch F, Naimi T, Enright MC (2003). Community-acquired methicillin-resistant Staphylococcus aureus carrying Panton-Valentine leukocidin genes: worldwide emergence. Emerg Infect Dis.

[3] Shittu AO, Udo EE, Lin J (2009). Phenotypic and molecular characterization of Staphylococcus aureus isolates expressing low- and high-level mupirocin resistance in Nigeria and South Africa. BMC Infect Dis.

[4] Goyal R, Das S, Mathur M (2022). Colonisation of methicillin resistant Staphylococcus aureus among health care workers in a tertiary care hospital of Delhi. Indian J Med Sci.

[5] Agarwal L, Singh AK, Agarwal A, Agarwal A (2016). Methicillin and mupirocin resistance in nasal colonizers coagulase-negative Staphylococcus among health care workers. Med J DY Patil Univ.

[6] Neela V, Ghasemzadeh Moghaddam H, van Belkum A, Horst-Kreft D, Mariana NS, Ghaznavi Rad E (2010). First report on methicillin-resistant Staphylococcus aureus of Spa type T037, sequence type 239, SCCmec type III/IIIA in Malaysia. Eur J Clin Microbiol Infect Dis.

[7] Shittu AO, Kaba M, Abdulgader SM, Ajao YO, Abiola MO, Olatimehin AO (2018). Mupirocin-resistant Staphylococcus aureus in Africa: a systematic review and meta-analysis. Antimicrob Resist Infect Control.

[8] (2022). Methicillin-resistant Staphylococcus aureus (MRSA) prevention tier 2 interventions. https://www.cdc.gov/infectioncontrol/pdf/strive/MRSA202-508.pdf.

[9] Nakajima J, Hitomi S, Kurihara Y (2011). Detection of methicillin-resistant Staphylococcus aureus with high-level resistance to mupirocin. J Infect Chemother.

[10] Farmer TH, Gilbart J, Elson SW (1992). Biochemical basis of mupirocin resistance in strains of Staphylococcus aureus. J Antimicrob Chemother.

[11] Patel JB, Gorwitz RJ, Jernigan JA (2009). Mupirocin resistance. Clin Infect Dis.

[12] Oommen SK, Appalaraju B, Jinsha K (2010). Mupirocin resistance in clinical isolates of staphylococci in a tertiary care centre in south India. Indian J Med Microbiol.

[13] (2023). Decolonization treatment for MRSA. https://www.health.wa.gov.au/Articles/A_E/Decolonisation-treatment-for-MRSA.

[14] Radhakrishna M, D'Souza M, Kotigadde S, Saralaya KV, Kotian MS (2013). Prevalence of methicillin resistant Staphylococcus aureus carriage amongst health care workers of critical care units in Kasturba Medical College Hospital, Mangalore, India. J Clin Diagn Res.

[15] (2022). Screening for MRSA carriers among health care workers in a tertiary care centre. http://www.ejournal-tnmgrmu.ac.in/index.php/medicine/article/view/17670.

[16] Kaur DC, Narayan PA (2014). Mupirocin resistance in nasal carriage of Staphylococcus aureus among healthcare workers of a tertiary care rural hospital. Indian J Crit Care Med.

[17] Mathanraj S, Sujatha S, Sivasangeetha K, Parija SC (2022). Screening for methicillin-resistant Staphylococcus aureus carriers among patients and health care workers of a tertiary care hospital in south India. Indian J Med Microbiol.

[18] Rongpharpi SR, Hazarika NK, Kalita H (2013). The prevalence of nasal carriage of Staphylococcus aureus among healthcare workers at a tertiary care hospital in Assam with special reference to MRSA. J Clin Diagn Res.

[19] Hudson IRB (1994). The efficacy of intranasal mupirocin in the prevention of staphylococcal infections: a review of recent experience. J Hosp Infect.

